# Safety and efficacy of ICI plus anlotinib vs. anlotinib alone as third-line treatment in extensive-stage small cell lung cancer: a retrospective study

**DOI:** 10.1007/s00432-021-03858-2

**Published:** 2021-11-19

**Authors:** Qing Chen, Yan Li, Wenjie Zhang, Chen Wang, Shengjie Yang, Qisen Guo

**Affiliations:** 1 Department of Oncology, Shandong Cancer Hospital and Institute, Shandong First Medical University and Shandong Academy of Medical Sciences, Jinan, 250117 China; 2 Weifang Medical University, Weifang, 261000 China

**Keywords:** Extensive-stage small cell lung cancer, Non-small cell lung cancer, Anlotinib, Immune checkpoint inhibitor, Safety, Efficacy

## Abstract

**Purpose:**

The objective of this study was to evaluate the safety and efficacy of immune checkpoint inhibitor (ICI) plus anlotinib as third-line treatment in extensive-stage small cell lung cancer (ES-SCLC).

**Methods:**

A total of 120 patients with ES-SCLC who were admitted to Shandong Cancer Hospital between January 2019 and December 2020 were retrospectively analyzed. They were divided into the observation group (*n* = 62) and the control group (*n* = 58) according to their different treatment plans. The observation group was given ICI plus anlotinib, while the control group was given anlotinib alone. The primary endpoint of the study was progression-free survival (PFS), and the secondary endpoints were the objective response rate (ORR) and disease control rate (DCR). An efficacy evaluation was carried out every 6 weeks. Univariate and multivariate analyses were performed to identify the prognostic factors. The main treatment-related adverse events were evaluated according to the Common Terminology Criteria for Adverse Events version 5.0.

**Results:**

In the observation group and the control group, the DCRs were 87.1% and 72.4% (*p* = 0.044), and the ORRs were 19.4% and 6.9% (*p* = 0.045), respectively. The median PFS was longer in the observation group (7.5 months) than in the control group (4.6 months) (*p* = 0.0033). In Cox regression analysis, the Eastern Cooperative Oncology Group performance status score, brain metastases and metastatic sites were prognostic factors of ICI plus anlotinib. Compared with the control group, grade 1–2 immune-related pneumonia and hypothyroidism of patients in the observation group were significantly increased (*p* < 0.05), but grade 3–4 treatment-related adverse reactions were not significantly increased (*p* > 0.05).

**Conclusion:**

ICI plus anlotinib showed promising efficacy and manageable toxicity in third-line treatment of ES-SCLC.

## Introduction

Small cell lung cancer (SCLC) accounts for 13–17% of all lung cancers, and smoking is the primary risk factor for SCLC (Oronsky et al. 2017; Govindan et al. 2006). SCLC has the characteristics of a high degree of malignancy and early metastasis. Therefore, the majority of patients are already in an extensive stage at the time of diagnosis (Kalemkerian 2016; Simon and Wagner 2003). Therapeutic options for extensive-stage small cell lung cancer (ES-SCLC) are limited. Chemotherapy plays an important role in the initial treatment of ES-SCLC. However, most patients develop recurrent disease after initial treatment, often with additional sites of metastasis (Ito et al. 2017). The emergence of targeted therapy and immunotherapy is promising for the treatment of SCLC.

Anlotinib is a small molecule oral multitargeted tyrosine kinase inhibitor that has the function of inhibiting angiogenesis and antitumor proliferation (Lin et al. 2018; Sun et al. 2016). It has been approved by the China National Medical Products Administration for ≥ third-line treatment of advanced SCLC (Cheng et al. 2018). Programmed death receptor 1/programmed death ligand 1 (PD-1/PD-L1) inhibitors are commonly used immune checkpoint inhibitors (ICIs). PD-1/PD-L1 inhibitors can not only restore the activity of T cells but also enhance the immune effect of T cells, thereby killing tumors (Efremova et al. 2018). A large number of studies have confirmed that PD-1/PD-L1 inhibitors can benefit ES-SCLC patients in terms of survival (Ready et al. 2019; Chung et al. 2020; Horn et al. 2018; Paz-Ares et al. 2019).

An increasing number of studies have shown that anlotinib and ICIs complement each other and play a synergistic role in antitumor therapy. First, anlotinib can normalize tumor blood vessels and improve the immune microenvironment of the tumor. In addition, the decrease in PD-L1 expressed by endothelial cells can cause an increase in VEGFR-2, indicating that PD-L1 has a potential regulatory effect on tumor angiogenesis (Jiang et al. 2015; Allen et al. 2017; Ramjiawan et al. 2017). Studies have confirmed that ICI plus anlotinib has a significant effect on the treatment of advanced NSCLC (Zhang et al., 2021; Liang and Wang 2019). Additionally, there was a study showing that anlotinib plus a PD-1 inhibitor may also be effective in the second-line or later treatment of relapsed SCLC (Zhang et al. 2021). Herein, we evaluated the efficacy and safety of ICI plus anlotinib versus anlotinib alone to find a high-efficiency and manageable-toxicity third-line treatment for ES-SCLC.

## Materials and methods

### Patients

We reviewed the electronic medical records of 120 patients with ES-SCLC who received anlotinib alone (*n* = 58) or ICI plus anlotinib (*n* = 62) for third-line treatment from January 2019 to December 2020 at Shandong Cancer Hospital, China. The inclusion criteria were as follows: (i) age at diagnosis between 18 and 75 years, (ii) the presence of pathologically or cytologically confirmed SCLC, (iii) the presence of imaging confirmed extensive stage, (iv) a prior lack of response or intolerance to two lines of treatment, (v) the presence of at least one measurable lesion as defined by the Response Evaluation Criteria in Solid Tumors (RECIST) version 1.1, (vi) an Eastern Cooperative Oncology Group performance status (ECOG PS) of 0–2, (vii) and no prior anlotinib or ICIs. Patients with NSCLC, uncontrolled hypertension, a bleeding tendency, ischemic cardiovascular disease, or severe liver and kidney dysfunction were excluded from the study.

A total of 58 patients received anlotinib alone. Anlotinib (Chia Tai Tianqing Pharmaceutical, China) was administered orally once daily (8 mg, 10 mg or 12 mg) on Days 1–14 of a 21-day cycle. A total of 62 patients received ICI plus anlotinib. Anlotinib (Chia Tai Tianqing Pharmaceutical, China) was administered orally once daily (8 mg, 10 mg or 12 mg) on Days 1–14 of a 21-day cycle. At the same time, the patients were treated with an ICI. The ICIs included sintilimab, toripalimab, camrelizumab, atezolizumab, nivolumab or durvalumab (Table 1). Tolerance and efficacy were evaluated every 6 weeks. The treatment was continued until disease progression, clinical deterioration, or unacceptable toxicity.

**Table 1:** Application of immune checkpoints

PD-1/PD-L1 inhibitor	Total
Sintilimab	18
Toripalimab	4
Camrelizumab	14
Atezolizumab	13
Nivolumab	4
Durvalumab	9

This study was approved by the ethics review board of the Shandong First Medical University and Shandong Academy of Medical Sciences. The requirement for informed consent was waived given the retrospective nature of the study.

### Evaluation of efficacy and adverse events

According to the Response Evaluation Criteria in Solid Tumors (RECIST) version 1.1 (Eisenhauer et al. 2009), an objective response rate (ORR) was defined as the sum of a complete response (CR) and a partial response (PR); disease control rate (DCR) was defined as the sum of CR, PR and stable disease (SD); PFS was calculated as the time from the initiation of treatment to progressive disease (PD) or death. Treatment-related adverse events (TRAEs) were divided into grades I–IV according to the Common Terminology Criteria for Adverse Events (CTCAE) version 5.0 (Freites-Martinez et al. 2021); the higher the grade, the worse the AEs.

### Statistical analysis

SPSS version 19.0 (IBM Corp., Armonk, New York) or GraphPad Prism 8.0 (La Jolla, California, United States) was used for all statistical analyses. The median PFS was calculated using the Kaplan–Meier method, and the survival curves were compared with the log-rank test. Cox proportional hazards regression models were used to analyze the correlation of baseline clinical characteristics with the efficacy of ICI plus anlotinib. A value of *p* < 0.05 was considered statistically significant.

## Results

### Patient characteristics

A total of 120 patients were included in this study. In the observation group, 38 (61.3%) were men and 24 (38.7%) were women. In the control group, 43 (74.1%) were men and 15 (25.9%) were women. All of them were aged 18–75. There were no significant differences in sex, age, smoking history, drinking history, ECOG PS, brain metastases, liver metastases, or metastatic sites between the two groups (*p* > 0.05). The baseline characteristics of the two groups are shown in Table 2.

**Table 2:** Baseline characteristics of two groups

Characteristics	Observation group	Control group	χ^2^	*p* value
Age, *n* (%)			0.000	0.994
≤ 65	47 (75.8%)	44 (75.9%)		
> 65	15 (24.2%)	14 (24.1%)		
Gender, *n* (%)			2.255	0.133
Male	38 (61.3%)	43 (74.1%)		
Female	24 (38.7%)	15 (25.9%)		
ECOG PS, *n* (%)			0.965	0.326
0–1	48 (77.4%)	49 (84.5%)		
2	14 (22.6%)	9 (15.5%)		
Smoking history, *n* (%)			0.072	0.789
Yes	25 (40.3%)	22 (37.9%)		
No	37 (59.7%)	36 (62.1%)		
Drinking history, *n* (%)			1.877	0.171
Yes	8 (12.9%)	13 (22.4%)		
No	54 (87.1%)	45 (77.6%)		
Liver metastases, *n* (%)			0.044	0.833
Absent	46 (74.2%)	44 (75.9%)		
Present	16 (25.8%)	14 (24.1%)		
Brain metastases, *n* (%)			0.109	0.741
Absent	27 (43.5%)	27 (46.6%)		
Present	35 (56.5%)	31 (53.4%)		
Metastatic sites, *n* (%)			0.058	0.810
≤ 3	35 (56.5%)	34 (58.6%)		
> 3	27 (43.6%)	24 (41.4%)		

*ECOG PS*: Eastern Cooperative Oncology Group Performance Status

### Efficacy

**Fig. 1:**
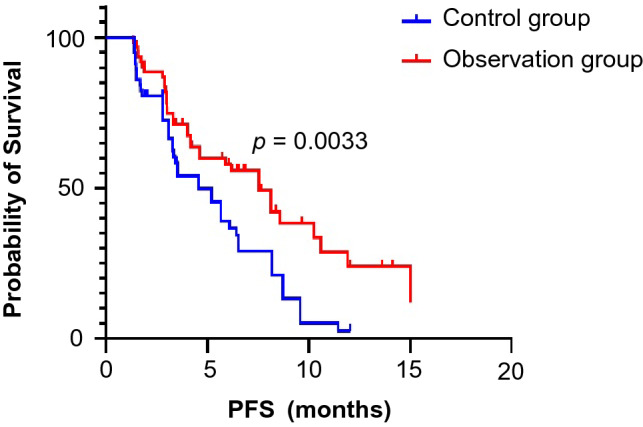
Kaplan–Meier survival curves of the progression-free survival (PFS) of the two groups

**Fig. 2:**
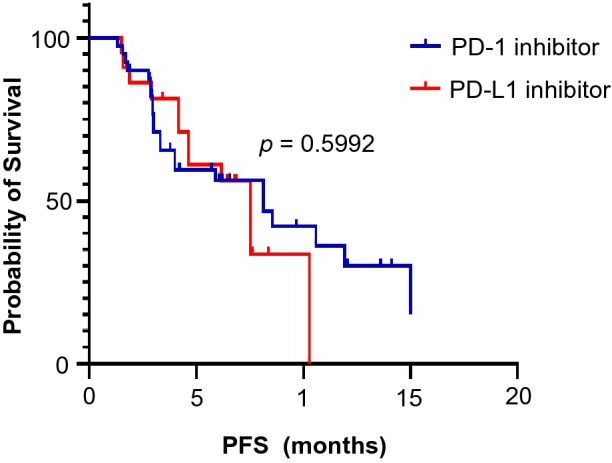
Kaplan–Meier survival curves for PFS were compared among the PD-1 inhibitor and PD-L1 inhibitor groups in the observation group

**Fig. 3:**
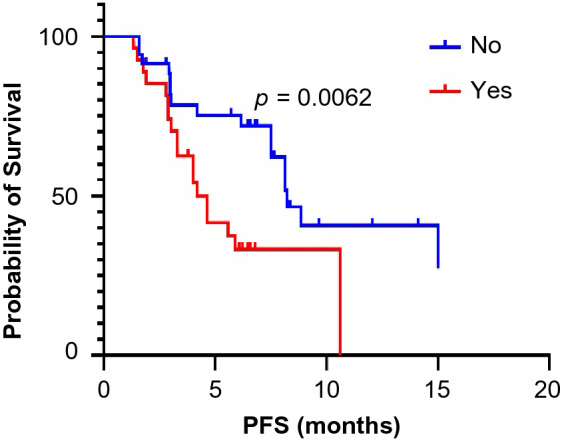
Kaplan–Meier survival curves for PFS were compared among the observation group with or without brain metastases

**Fig. 4:**
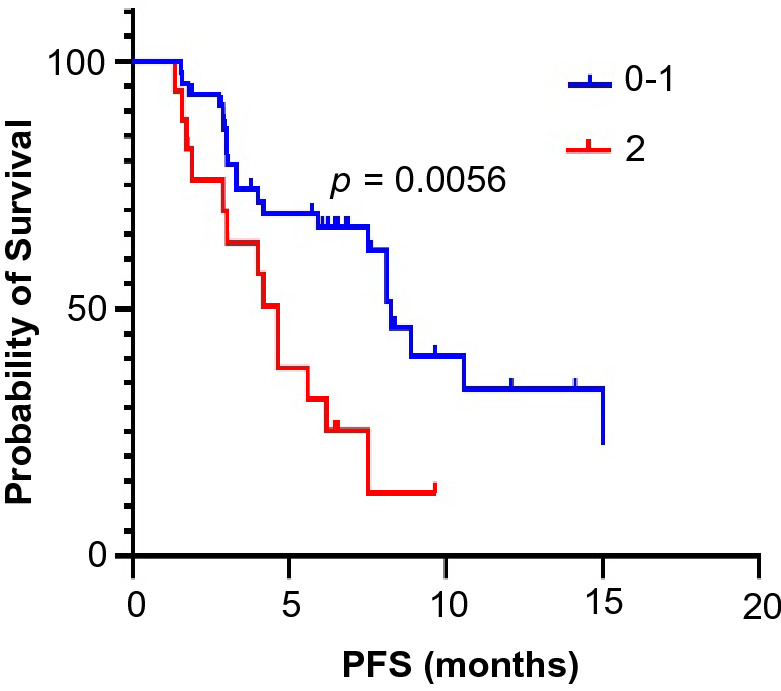
Kaplan–Meier survival curves for PFS were compared among the observation groups with different Eastern Cooperative Oncology Group performance statuses

**Fig. 5:**
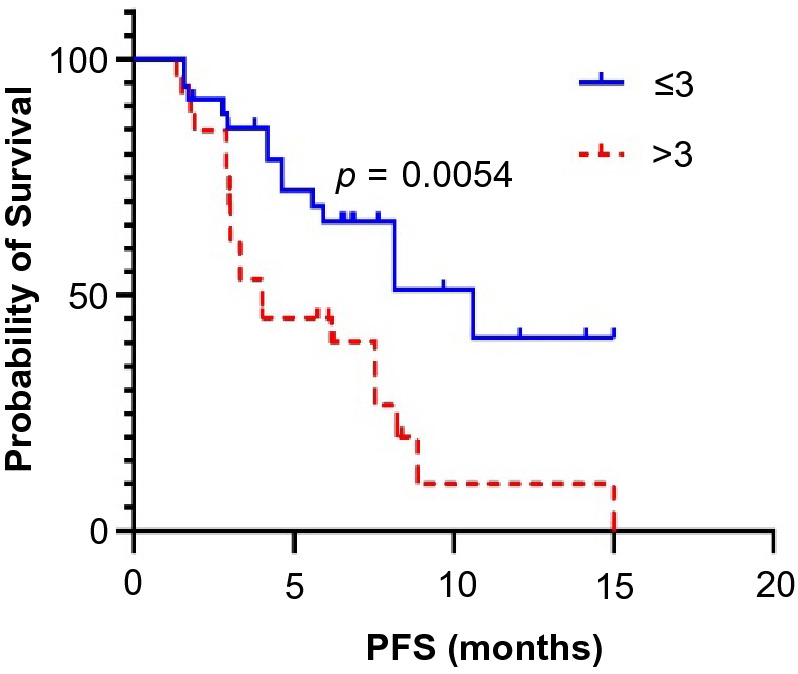
Kaplan–Meier survival curves for PFS were compared among the observation groups with different metastatic sites

All patients received at least 6 weeks of treatment and follow-up. The ORR of the observation group was higher than that of the control group (19.4% vs. 6.9%, *p* = 0.045). The DCRs of the observation group and the control group were 87.1% and 72.4%, respectively (*p* = 0.044) (Table 3). As exhibited in Fig. 1, the median PFS of the observation group was 7.5 months (95% CI 5.5–9.6 months) and that of the control group was 4.6 months (95% CI 3.0–6.2 months, *p* = 0.0033). In the observation group, the median PFS of patients treated with PD-1 inhibitors was not significantly different from that of patients treated with PD-L1 inhibitors (8.1 vs. 7.5 months, *p* = 0.5992) (Fig. 2). Through univariate and multivariate analysis, we found that sex, age, smoking history, drinking history and liver metastases had no influence on the median PFS of ICI plus anlotinib, but ECOG PS, brain metastases and metastatic sites were reliable factors of prognosis (Table 4). As presented in Figs. 3, 4 and 5, among all patients in the observation group, the median PFS of patients without brain metastases was 7.5 months, and the median PFS of patients with brain metastases was 4.6 months (*p* = 0.0062). Patients who had an ECOG PS of 0–1 had a significantly higher median PFS than patients with an ECOG PS of 2 (8.2 vs. 4.6 months, *p* = 0.0056). Patients with less than or equal to three metastatic sites had a higher PFS than those with more than three metastatic sites (10.6 vs. 4.0 months, *p* = 0.0054).

**Table 3:** Short-term efficacy in the two groups of patients

Group	CR	PR	SD	PD	ORR	*p* value	DCR	*p* value
Observation group	2	10	42	8	12 (19.4%)	0.045	54 (87.1%)	0.044
Control group	0	4	38	16	4 (6.9%)		42 (72.4%)

**Table 4:** Univariate and multivariate cox regression analysis of factors associated with PFS

Characteristics	Univariate analysis		Multivariate analysis	
	HR 95% CI	*p* value	HR 95% CI	*p* value
Age (≤ 65 vs. > 65)	0.827–5.036	0.122		NI
Gender (male vs. female)	0.638–2.583	0.483		NI
Smoking history (yes vs. no)	0.374–1.531	0.437		NI
Drinking history (yes vs. no)	0.262–1.549	0.320		NI
ECOG PS (0–1 vs. 2)	0.185–0.777	0.080	0.197–0.871	**0.004**
Liver metastases (yes vs. no)	0.410–1.625	0.563		NI
Brain metastases (yes vs. no)	0.178–0.778	**0.009**	0.187–0.903	**0.027**
Metastatic sites (≤ 3 vs. > 3)	0.196–0.781	**0.008**	0.172–0.712	**0.002**

Boldness indicates *p* value less than 0.05

*ECOG PS* Eastern Cooperative Oncology Group Performance Status, *HR* hazard ratio, *CI* confidence interval, *NI* not included in multivariate model

### Safety

Eleven patients in the observation group and 0 in the control group experienced grade 1–2 hypothyroidism (*p* = 0.002). In addition, between-group significant differences were also seen for grade 1–2 immune-related pneumonia (11.3% in the observation group vs. 0% in the control group, *p* = 0.008). There were no significant differences between the two groups for grade 3–4 AEs. The key grade 3–4 AEs in the observation group were hypertension (*p* = 0.808), bone marrow suppression (*p* = 0.168), vomiting/diarrhea (*p* = 0.168), gingival bleeding (*p* = 0.331), gastrointestinal bleeding (*p* = 0.331), hypothyroidism (*p* = 0.168), hand–foot syndrome (*p* = 0.526) and immune-related pneumonia (*p* = 0.090). Six patients reduced the dose of anlotinib due to TRAEs in the control group. Treatment discontinuation occurred in two patients, and drug reduction occurred in ten patients due to TRAEs in the observation group. No patients in either group experienced treatment-related death (Tables 5 and 6).

**Table 5:** Treatment-related adverse events in the two groups (*n* %)

AEs	Grades 1–2		*p* value	Grades 3–4		*p* value
	Control group	Observation group		Control group	Observation group	
Hypertension	10 (17.2%)	12 (19.3%)	0.765	4 (6.9%)	5 (8.1%)	0.808
Bone marrow suppression	5 (8.6%)	9 (14.5%)	0.315	0 (0.0%)	2 (3.2%)	0.168
Transaminitis	0 (0.0%)	2 (3.2%)	0.168	0 (0.0%)	0 (0.0%)	–
Fatigue	3 (5.2%)	7 (11.3%)	0.226	0 (0.0%)	0 (0.0%)	–
Rash	0 (0.0%)	3 (4.8%)	0.090	0 (0.0%)	0 (0.0%)	–
Vomiting/diarrhea	5 (8.6%)	6 (9.7%)	0.841	0 (0.0%)	2 (3.2%)	0.168
Cough	2 (3.4%)	1 (1.6%)	0.520	0 (0.0%)	0 (0.0%)	–
Hand–foot syndrome	8 (13.8%)	11 (17.7%)	0.554	3 (5.2%)	5 (8.1%)	0.526
Gingival bleeding	5 (8.6%)	6 (9.7%)	0.841	0 (0.0%)	1(1.6%)	0.331
Gastrointestinal bleeding	2 (3.4%)	3 (4.8%)	0.703	0 (0.0%)	1 (1.6%)	0.331
Hypothyroidism	0 (0.0%)	11 (17.7%)	**0.002**	0 (0.0%)	2 (3.2%)	0.168
Immune-related pneumonia	0 (0.0%)	7 (11.3%)	**0.008**	0 (0.0%)	3 (4.8%)	0.090

Boldness indicates *p* value less than 0.05

*AEs* adverse events

**Table 6:** Treatment administration and dose modification of anlotinib

	Dose of anlotinib	
	Observation group	Control group
Initial dosage (mg)		
8	8	6
10	17	10
12	37	42
Modification of dosage (mg)		
10 → 8	3	2
12 → 10	6	3
12 → 8	1	1

## Discussion

The findings of the retrospective study proved that anlotinib combined with ICI is a promising regimen for the third-line treatment of patients with ES-SCLC. Although Group 1–2 hypothyroidism and immune-related pneumonia were significantly higher under anlotinib plus ICI, no treatment-related deaths occurred. ICI plus anlotinib is safe and tolerable.

In our study, the results for the control group were slightly higher than the findings of the ALTER1202 trial (Cheng et al. 2018), which assessed the efficacy and safety of anlotinib as a third-line or further-line in relapsed SCLC. The results of the ALTER1202 trial showed that the ORR (4.94% vs. 2.63%, *p* = 1.0000), DCR (71.60% vs. 13.16%, *p* < 0.0001) and median PFS (4.1 vs. 0.7 months, *p* < 0.0001) were higher in the anlotinib group than in the placebo group. In our study, the ORR (6.9%), DCR (72.4%) and PFS (4.6 months) of the anlotinib group were higher than those in the ALTER1202 study. The reason for this may be that anlotinib was only used as the third-line treatment in our study while it was used not only as third-line treatment but also as further-line treatment in the ALTER1202 trial.

Although a large number of studies have proven that ICIs are effective in the treatment of SCLC, the appropriate time for the use of ICIs is uncertain. Studies have shown that PD-L1 inhibitors and PD-1 inhibitors are effective in the first-line treatment of ES-SCLC (Horn et al. 2018; Liu et al. 2019; Paz-Ares et al. 2018; Leal et al. 2020). However, PD-1/PD-L1 inhibitors failed to demonstrate significant efficacy in maintenance therapy and second-line treatment after first-line treatment of ES-SCLC (Pujol et al. 2019; Owonikoko et al. 2019; Spigel et al. 2021; Goldman et al. 2018; Gadgeel et al. 2018). PD-1 inhibitors have been proven useful in third-line and more treatment of ES-SCLC. The results of the CheckMate-032 study subgroup analysis showed that nivolumab as a single agent of the third line and above treatment for SCLC had an ORR of 11.9% and a median duration of response (DOR) of 17.9 months. The median PFS was 1.4 months (95% CI 1.3–1.6 months), and the incidence of grade 3–4 AEs was 11.9% (Ready et al. 2019). This suggests that nivolumab is long lasting and well tolerated as a third line and above treatment for SCLC. The KEYNOTE028/158 study analyzed the efficacy of another PD-1 inhibitor, pembrolizumab, in the third line and above treatment of SCLC. The results showed that the ORR of pembrolizumab was 19.3% (95% CI 11.4–29.4%), the PFS was 2.0 months (95% CI 1.9–3.4 months), and the median OS was 7.7 months (95% CI 5.2–10.1 months) (Chung et al. 2020). However, the efficacy of PD-L1 inhibitors after two or more lines of previous therapy in patients with ES-SCLC remains unknown. In view of the large difference in efficacy of PD-1 inhibitors and PD-L1 inhibitors in the treatment of SCLC and the uncertainty of the timing of their use, we analyzed PD-1 inhibitors and PD-L1 inhibitors in separate groups. In our study, the median PFS in the PD-1 inhibitor group was 8.1 months and that in the PD-L1 inhibitor group was 7.5 months (*p* = 0.5992). Hence, regardless of whether anlotinib was combined with a PD-1 inhibitor or PD-L1 inhibitor, there was no significant difference in median PFS.

Brain metastasis is considered to be one of the factors affecting the prognosis. In the observation group, the median PFS of patients without brain metastases was significantly longer than that of patients with brain metastases (*p* = 0.0062). The incidence of brain metastases from lung cancer is the highest of all tumors, and more than 25% of patients develop brain metastases during the course of the disease (Zimm et al. 1981; Sheehan et al. 2002). In established brain metastases, the tumor microenvironment is comprised of the innate immune system, namely, microglia and macrophages, and adaptation to the immune system is mainly achieved through T cells. On the one hand, brain metastases can manipulate the metabolites and matrix components in their microenvironment to influence the immune response. On the other hand, brain metastases can regulate and activate the function of microglia and produce inducible nitric oxide synthase and tumor necrosis factor-α, which can lyse target cells (Holmgaard et al. 2015; Munn and Mellor 2016; He et al. 2006). There are prerequisites for a response to immunotherapy in the microenvironment of brain metastases. In addition, the efficacy of anlotinib in patients with brain metastases was proven by the ALTER 1202 trial subgroup analysis, which showed that the PFS of patients with brain metastases was prolonged by 3 months (3.8 vs. 0.8 months, HR 0.15), and OS was prolonged by 3.7 months (6.3 vs. 2.6 months, HR 0.23) (Cheng et al. 2019). Therefore, for ES-SCLC patients with brain metastases, we recommend the use of anlotinib combined with ICIs as third-line treatment.

ECOG PS is also one of the independent factors affecting the therapeutic effect of anlotinib plus ICI. The results showed that the median PFS in patients with ECOG PS of 0–1 was much longer than that in patients with ECOG PS of 2 (*p* = 0.0056), which is consistent with a meta-analysis of SCLC at different stages. The meta-analysis showed that patients with a PS score of 0–1 have the best prognosis, and patients with a PS score of ≥ 2 have the worst prognosis (Foster et al. 2009). The higher the ECOG PS, the shorter the survival period (Reck et al. 2012). Therefore, when the physical condition is good and the ECOG PS score is low, ICI plus anlotinib shows promising efficacy as the third-line treatment of ES-SCLC.

In addition, the number of metastatic sites is also one of the nonnegligible factors that affect the efficacy of ICIs plus anlotinib. In our study, the median PFS of patients with less than three metastases was higher than that of patients with more than three metastases (10.6 vs. 4.0 months, *p* = 0.0054). This result suggests that the lower the number of metastatic sites, the better the efficacy of ICI plus anlotinib.

However, our study also has shortcomings. Due to the nature of retrospective research, selection bias is inevitable and may result in lower reliability of the judgment of comprehensive safety or efficacy of both regimens. In addition, due to the different physical conditions of the patients and the small sample size, we could not analyze the different doses of anlotinib between the groups. Furthermore, other outcomes, such as OS and health-related quality of life, were not analyzed in this study.

In general, anlotinib plus ICI can achieve promising survival outcomes among patients with ES-SCLC, and the TRAEs are safe and tolerable. Brain metastases, metastasis sites and ECOG PS are factors affecting the prognosis. The therapeutic effect was equivalent whether anlotinib was combined with a PD-1 inhibitor or a PD-L1 inhibitor.

## Data Availability

Datasets generated and analyzed during the study are available from QC on reasonable request.
